# Involvement of Suppressive B-Lymphocytes in the Mechanism of Tolerogenic Dendritic Cell Reversal of Type 1 Diabetes in NOD Mice

**DOI:** 10.1371/journal.pone.0083575

**Published:** 2014-01-17

**Authors:** Valentina Di Caro, Brett Phillips, Carl Engman, Jo Harnaha, Massimo Trucco, Nick Giannoukakis

**Affiliations:** 1 Division of Immunogenetics, Department of Pediatrics, University of Pittsburgh School of Medicine, Pittsburgh, Pennsylvania, United States of America; 2 RiMed Foundation, Palermo, Italy; 3 Penn State University Hershey Medical Center, Hershey, Pennsylvania, United States of America; 4 Department of Pathology, University of Pittsburgh School of Medicine, Pittsburgh, Pennsylvania, United States of America; Children's Hospital Boston/Harvard Medical School, United States of America

## Abstract

The objective of the study was to identify immune cell populations, in addition to Foxp3+ T-regulatory cells, that participate in the mechanisms of action of tolerogenic dendritic cells shown to prevent and reverse type 1 diabetes in the Non-Obese Diabetic (NOD) mouse strain. Co-culture experiments using tolerogenic dendritic cells and B-cells from NOD as well as transgenic interleukin-10 promoter-reporter mice along with transfer of tolerogenic dendritic cells and CD19+ B-cells into NOD and transgenic mice, showed that these dendritic cells increased the frequency and numbers of interleukin-10-expressing B-cells *in vitro* and *in vivo*. The expansion of these cells was a consequence of both the proliferation of pre-existing interleukin-10-expressing B-lymphocytes and the conversion of CD19+ B-lymphcytes into interleukin-10-expressing cells. The tolerogenic dendritic cells did not affect the suppressive activity of these B-cells. Furthermore, we discovered that the suppressive murine B-lymphocytes expressed receptors for retinoic acid which is produced by the tolerogenic dendritic cells. These data assist in identifying the nature of the B-cell population increased in response to the tolerogenic dendritic cells in a clinical trial and also validate very recent findings demonstrating a mechanistic link between human tolerogenic dendritic cells and immunosuppressive regulatory B-cells.

## Introduction

Re-establishment of peripheral tolerance has long been sought as a method to effectively treat type 1 diabetes mellitus (T1D) especially new-onset disease, since functional beta cell mass can persist for months to years after clinical diagnosis. T1D is an autoimmune disease due to the loss of insulin production consequent to a chronic inflammation-driven impairment and eventual eradication of a critical mass of pancreatic beta cells [1], [2], [3]. Studies conducted in non-obese diabetic (NOD) mice, a strain that spontaneously develops diabetes of an autoimmune nature, have highlighted the critical role of the adaptive immune response in the pathogenesis of the disease [4], [5].

It is now clear that the targeting of insulin by the immune system, the underlying immune hallmark of T1D etiopathogenesis, is a result of central (thymic) and peripheral tolerance failure [6]. Central failure is a consequence of impaired expression and presentation of proinsulin to nascent thymocytes by medullary thymic epithelial cells [7], [8]. Peripheral failure is a chronic process characterised by pathologic changes to regulatory immune cell populations in the secondary lymphoid organs [9], [10], [11], [12], [13]. Rebalancing the regulatory∶effector immune cell numbers in the periphery by taking advantage of homeostatic expansion of populations like T-cells and B-cells secondary to their deletion using antibodies, for example, has resulted in attenuation of a variety of experimental autoimmunity in mice and rats, including remission of T1D [14], [15], [16]. The preservation of residual beta cell mass and, in some instances, the temporary withdrawal of insulin treatment in new onset T1D subjects, believed to be due to the rebalancing of the Foxp3+ T-cell∶effector T-cell population following the depletion of T-cells using a CD3-targeting antibody, is one of the modern examples where restoration of peripheral tolerance was successfully, even though transiently, achieved [17], [18], [19].

In addition to Foxp3+ regulatory T-cells, DC are key regulators of peripheral tolerance making them promising therapeutic vehicles to treat autoimmunity including T1D. Key general features of DCs that activate and maintain immunosuppressive states include: low antigen-presentation capacity (low levels of class I and class II MHC expression on the cell surface), low-to-absent co-stimulation ability, poor to absent allostimulatory ability to induce T-cell proliferation in allogeneic mixed leukocyte culture or in antigen-specific recall responses, production of TH2-type cytokines and retinoic acid [20], [21]. We have shown that administration of DC treated *ex vivo* with short double-stranded NF-kappaB oligonucleotide decoys [22] and antisense oligonucleotides targeting CD40, CD80 and CD86 can prevent T1D in the non-obese diabetic (NOD) mouse strain [23] and can prolong allograft survival [24]. Administration of these immunosuppressive DC promotes production of cytokines that impair T-cell activation and is associated with increased numbers and enhanced function of Foxp3+ T regulatory (Treg) cells [reviewed in [25], [26]]. Indeed, the success of suppressive DC-based therapies to upregulate Foxp3+ Tregs are particularly striking in diabetic mouse models [27], [28]. In addition to the reproducible effects of DC on Treg upregulation, emerging data indicate that other immunoregulatory cells, including NKT [29] and B-lymphocytes [30] are DC-senstitive in their role of maintaining/promoting tolerance.

The involvement of B-lymphocytes in the etiopathogenesis of T1D was first uncovered in the NOD mouse strain, where mice deficient in B-lymphocytes as a consequence of IgM mutation, or treatment with anti-IgM antibodies exhibited significant protection from the disease [31], [32]. Most studies suggested that the pathogenic role of B-lymphocytes lies largely in their ability to act as antigen-presenting cells [33], [34], [35], [36], [37], [38], producers of autoreactive antibodies [39], [40] and modulators of the type of T-cells that enter and are active within the pancreatic and islet environment [41]. Most importantly, B-lymphocyte depletion, by anti-CD20, anti-CD45RB, and anti-CD22 antibodies, resulted in the sustained and stable prevention and, in some instances, the reversal of T1D in NOD mice [42], [43], [44], [45], [46], [47] as well as facilitation of islet allograft survival in NOD mice [44]. Indeed, efficacy of the anti-CD20 antibody treatment in NOD mice underlay the Rituximab clinical trial in new-onset human patients [48], [49], [50]. These seemingly disparate observations were recently reconciled with the identification of one or more B-lymphocyte populations that are inherently immunosuppressive, whose frequency and, possibly activity, may change over time and during perturbations in peripheral tolerance [30], [51].

Immunosuppressive B-cells, widely referred to as B-regulatory cells (Bregs) in mice exist in the CD1d^HIGH^ CD5+ IL-10-producing population. These cells can suppress experimental colitis, arthritis and lupus [52]. Adoptive transfer of LPS-stimulated B cells prevented T1D development in NOD mice [53], while CD40 antibody-stimulated B cells prevented arthritis [54]. In humans, in addition to the IL-10-producing CD1d+ CD5+ B-cells [termed “B10 Bregs”; [52], [55]], CD19+ CD24^HIGH^ CD27+ CD38^HIGH^ B-cells are also suppressive, relying partly on IL-10 [56].

We have established a protocol to generate stably-immunosuppressive, tolerogenic DC ex vivo from peripheral blood mononuclear cells (PBMC) [57]. These cells are products of DC progenitors generated in the presence of antisense DNA targeting the primary transcripts of CD40, CD80 and CD86. Administration into established adult T1D subjects resulted in an increase in the frequency of a B-cell population that suppressed proliferation of syngeneic T-cells in response to allostimulation in vitro [57]. Of note, these B-cells did not rely on IL-10 for suppressive ability. More recently, we confirmed that these suppressive B-cells were essentially-identical in phenotype to one population of human Bregs [56], [58], [59], [60] and that co-culture with co-stimulation-impaired DC *in vitro* resulted in increased proliferation *in vitro* [61]. Given our observations with the human suppressive B-cell population and its response to the co-stimulation-impaired, tolerogenic DC, we hypothesised that murine Bregs would be similarly impacted by exposure to the murine embodiment of these human tolerogenic DC. To address this hypothesis, we aimed to demonstrate a direct effect of the tolerogenic DC on increasing Breg numbers either through expansion of existing cells (proliferation) and/or differentiation of B-cells into Bregs. Concurrently, this study was also aimed at a first understanding of the potential mechanisms by which the tolerogenic DC could promote Breg expansion, with a specific focus on retinoic acid.

Herein, as we affirm the ability of co-stimulation-deficient, tolerogenic DC to reverse new onset T1D in NOD mice, we demonstrate that the frequency of IL-10+ B-cells increases, largely by differentiation of CD19+ B-cells, when exposed to tolerogenic DC *in vitro* and *in vivo*. Even though CD1d+ CD5+ IL-10+ B-cells [51], [55] comprise the majority of the suppressive population expanded in response to the tolerogenic DC, our data suggest that IL-10 is not a *conditio sine qua non* for suppression of T-cell proliferation to allostimulation *in vitro*. The suppression seems to require physical contact between B-cells and T-cells. We also report that B10 Bregs express retinoic acid (RA) receptors. Importantly, our tolerogenic DC express aldehyde dehydrogenase (ALDH), the rate-limiting enzyme for retinoic acid biosynthesis, and produce RA *in vitro*. Taken together, our findings point to a novel mechanism of DC-mediated suppression of T1D, involving the induction of suppressive B-cells, including IL-10+ Bregs. Such suppressive B-cells could in principle be readily manipulated *ex vivo* or *in vivo* to promote tolerance to T1D and perhaps other autoimmune conditions as an alternative, or as an additive approach to tolerogenic DC.

## Materials and Methods

### Animals

#### Ethics Statement on Animal Use

This study was carried out in strict accordance with the recommendations in the Guide for the Care of Animals of the National Institutes of Health. The protocols were approved by the IACUC of the University of Pittsburgh (Protocol numbers 1110982 and 1112140). All procedures and euthanasia were conducted according to these approved protocols with an aim to ameliorate and potential animal discomfort.

Female NOD/LtJ mice were purchased from Jackson Laboratories (Bar Harbor, ME) and were used between the ages of 8–18 weeks or when confirmed diabetic (two consecutive readings of tail vein blood glucose >300 mg/dL). C57BL/6 transgenic mice expressing GFP under the control of the IL-10 promoter (IL-10 GFP “knock-in”; IL10^gfp^; [62] were purchased from the Jackson Laboratories and maintained as a colony and along with the transgenic control strain wild-type C57BL/6 female mice (Jackson Laboratories), they were used between the ages of 7–12 weeks. All mice were maintained in a specific pathogen-free environment in the Animal Facility of the Rangos Research Center in accordance with institutional, state and federal guidelines. All animal experiments were conducted following approved protocols by the University of Pittsburgh IACUC.

### Generation of murine bone marrow-derived DC and administration in vivo

DC (control DC or immunosuppressive DC; cDC and iDC, respectively) were generated *in vitro* from bone marrow progenitors using previously-published methods [23], [63]. cDC and iDC were administered subcutaneously (s.c.) into the abdominal flank overlying the predicted anatomical location of the pancreas. DC were routinely administered at 1–2×10^6^ cells in 150–200 microliters volume sterile, endotoxin-free PBS. Mice that were confirmed to be diabetic, were immediately given between 0.5–2 units of a Novolin∶Humulin (1∶1) mixture i.p. once daily until non-fasting blood glucose fell to below 280 mg/dL. From the time of diabetes confirmation, only mice that returned to non-fasting glucose <280 mg/dL within a 15–20 day window of daily insulin administration were considered for cDC or iDC treatment. On the first day where the glucose reading was <280 mg/dL, insulin was stopped and cDC or iDC were immediately administered. Mice were given either a single injection or multiple injections (eight injections) of cDC or iDC in PBS vehicle. In the multiple injection recipients, cDC/iDC were administered once weekly for a period of eight weeks. No insulin was given during the DC treatment or at any time thereafter. Glucose monitoring continued twice weekly for a period of 30 days, and then once weekly for at least 6 months when normoglycemic mice were euthanized. Mice that returned to diabetes at any time after the DC treatment were euthanized. An additional control group of mice were treated with insulin to normalise glucose and then insulin was withdrawn. These mice were euthanised upon return of hyperglycemia.

### Multiparameter/multicolor flow cytometry and flow sorting

We used a FACSCalibur/FACSAria with DIVA support (BD Biosciences) with species-specific antibodies, non-overlapping fluorophores and appropriate isotype controls for flow-sorting and flow cytometry. With the exception of experiments where the ALDEFLUOR reagent was used (see further below), in all measurements of DC populations, Fc-Block (BD Biosciences) was used prior to antibody staining. Cells were antibody-stained either after pre-enrichment for specific populations over magnetic columns (e.g. CD3+ T-cells or CD19+ B220+ B-cells; MIltenyi Biotec), or stained as freshly-isolated single cells from spleen and/or pancreatic lymph nodes or from cultured cells *in vitro*. The antibodies used for flow cytometry and/or flow sorting (all from BD Biosciences) were - B220 (clone RA3-6B2), CD19 (clone 1D3), CD27 (clone M-T271), CD38 (clone 90), CD38 (clone HIT2), CD5 (clone 53-7.3), CD1d (clone 1B1), IgD (clone 11.26c.2a), IgM (clone R6-60.2), CD40 (clone 3/23), IL-10 (intracellular; clone JES5-16E3). The antibody specific for the CD21/CD35 complex (clone 8D9) was purchased from eBioscience. As part of DC characterisation by flow cytometry, the CD11c antibody (clone HL3) was used.

### Isolation of T- and B-cell populations

Freshly-isolated spleen and, where indicated, pancreatic lymph nodes (PLN) were used. CD3+ T-cells were enriched from freshly-collected spleen by passing splenocytes over columns (R&D Systems). B-cells were enriched from the splenocytes by column-assisted positive selection into CD19+ cells (Miltenyi Biotec). To further enrich B-cells into those that produced IL-10, two methods were pursued depending on the experiment. Method 1 involved a first step enrichment of splenocytes from freshly-collected spleen into IL-10-producing cells using the IL-10 Secretion Assay Cell Enrichment & Detection Kit (Cat# 130-090-490, Miltenyi Biotec). These cells were then further enriched into B220+ CD19+ cells by positive selection over specific selection columns. In Method 2, IL-10 producing splenocytes were first isolated from freshly-collected spleen using the selection kit referred to above. These cells were then stained with fluorescence-tagged antibodies and flow sorted into more homogeneous cell populations depending on the experiment. All antibodies were purchased from BD Biosciences.

IL-10-expressing CD1d+ CD5+ CD19+ regulatory B-cells (“B10 Bregs”) were characterised and flow-sorted as described [52], [55]. Where B10 Bregs were used in functional studies *in vitro*, they were first enriched into CD19+ B220+ cells using magnetic isolation columns, followed by passage through the IL-10 pre-enrichment column and then further flow-sorted into CD1d+ CD5+ cells.

### Microscopy for B10 Bregs

Cytospins of flow-sorted B10 Bregs from freshly-collected splenocytes of NOD female mice at 10 weeks of age were processed for hematoxylin and eosin staining. Images were captured by a Zeiss Axioplan2 microscope at 63× magnification.

### Ascertainment of suppressive B-cell frequency in vivo in DC recipients

1–2×10^6^ cDC, iDC, or PBS vehicle were injected s.c. into mice into the abdominal region overlying the predicted anatomical location of the pancreas. Three days following DC injection, spleens were harvested and processed into single cells. CD19+ B-cell as well as B10 Breg frequency was measured by flow cytometry. For some *in vitro* experiments (e.g. allogeneic mixed leukocyte reactions (MLR)), cells from identically-treated mice were flow sorted into B10 Bregs or B220+ CD19+ IL-10+ CD11c− B-cells from IL-10-producing, pre-enriched B-cells prior to co-culture or further characterisation.

### Proliferation of IL-10-expressing B-cells and conversion of B-cells into IL-10-expressing cells in vitro

B-cells were enriched from splenocytes of IL10^gfp^ transgenic mice; splenocytes from these transgenic mice were first enriched into CD19+ B220+ CD11c− GFP-negative or CD19+ B220+ CD11c− GFP-positive cells by sequential selection over commercially-available magnetic columns (Miltenyi Biotec) and then flow sorted to purity into GFP+ or GFP− cells. For flow cytometry and flow sorting, stringent control for B-cell autofluorescence was conducted by establishing the cell selection gate using B-cells from the wild-type strain of the transgenic as well as very stringent gating to remove autofluorescent cells co-incident inside the GFP+ cell populations. All cells whose fluorescence in the GFP channel overlapped with that of the non-transgenic B-cells were considered GFP− cells. The GFP− and GFP+ cells were seeded in triplicate at 5×10^4^–1×10^5^ cells in the presence of an equal number of cDC or iDC, or in medium alone. Five days later, the frequency of GFP+ and GFP− cells was ascertained by flow cytometry.

### Proliferation of T-cells in vitro

Freshly-isolated splenocytes were irradiated (2800 rad) in preparation for MLR co-cultures. Cells were plated as phenotypically-homogeneous populations (splenocytes alone, T-cells alone, B-cells alone) or in co-culture with the following cell numbers per well: Splenocytes at 2×10^5^ cells, T-cells at 2×10^5^ cells, and B-cells at 2×10^4^–2×10^5^ cells. IL-10 neutralising antibody at a 1 microgram/ml final concentration (eBiosciences) was added to some co-cultures to determine the role of IL-10 in suppression. On day 4 post-plating, BrdU was added to individual wells to a final concentration of 1 mM. T-cell proliferation was measured with the FITC BrdU Flow Kit (BD Biosciences) on day 5 by flow cytometry.

To determine if soluble mediators were involved in B-cell-induced suppression, allogeneic MLR co-cultures with splenic or PLN cells were re-established in HTS Transwell – 96 Well Permeable Plates with a membrane pore size of 0.4 micrometers (Corning). Flow-sorted B-cells were added into the Transwell system at a total of 1.25×10^5^ cells per well. B-cells were plated onto the Transwell membrane, while T-cells and splenocytes were co-cultured in the bottom chamber, below the insert. Proliferation was ascertained by BrdU incorporation using flow cytometry.

### Gene expression

B10 Bregs and B220+ CD19+ IL-10+ CD11c− B-cells were flow-sorted from freshly-isolated splenocytes to >95% purity. Total mRNA was obtained using the MACS mRNA Isolation System (Miltenyi Biotec) in preparation for RT-PCR. cDNA was synthesised using the SuperScript III System (Invitrogen) and then real-time PCR to amplify retinoic acid receptor (RAR) alpha and retinoid X receptor (RXR) cDNA was conducted with the iQ SYBR Green Mix (BioRad) in an iCycler. Relative steady-state mRNA levels were calculated based on the 2***^Δ-ΔCt^*** method after correction for *GAPDH* housekeeping gene expression levels.

Primer sequences used (adopted from [64])

*RAR alpha*: forward: 5′-AGGCCATCACAACTACCTGC-3′

reverse: 5′-GGAAAGAAGAAGGCGTAGGG-3′

*RXR*:

forward: 5′-CTTTGACAGGGTGCTAACAGAGC-3′

reverse: 5′-ACGCTTCTAGTGACGCATACACC-3′

*GAPDH (housekeeping control):*

forward: 5′-GGGTGGAGCCAAACGGGTC-3′

reverse: 5′-GGAGTTGCTGTTGAAGTCGC-3′

*Ascertainment of RA production by DC in vitro and flow sorting of RA-producing cells.*

Complementary methods were used to detect and confirm RA production by DC. In the first, we used the ALDEFLUOR reagent (StemCell Technologies), a substrate of ALDH [65], [66] to flow sort RA-producing CD11c+ cells. In some instances, cells were co-stained with antibodies for specific cell surface proteins in order to measure specific populations by flow cytometry or to flow sort along RA positivity. To confirm RA production, we co-cultured candidate DC populations with HEK293 cells transiently-transfected with a luciferase reporter under the control of a minimal promoter with multiple RA response elements (SABiosciences), where the DC and the transfected cells were separated by Transwell. The DC were placed on the upper chamber membrane while the transfected reported cells were placed in the lower plate wells. DC were co-cultured with the reporter cells for 24–72 hours and the level of luciferase activity (Dual Luciferase Assay; Promega) was used as an indirect measure of bioactive RA production by DC. Appropriate controls included untransfected cells, reporter cells alone, medium alone and DC only. Additionally, we also conducted the experiments using dual-reporter HEK293 cells where two plasmids were co-transfected (Renilla-Luc as the transfection control and firefly-Luc under the control of the RA respnse element minimal promoter).

### Statistical considerations

Statistically-relevant differences among means (Student's t-test, ANOVA) or medians (Kruskal-Wallis test) were ascertained using GraphPad Prism version 4 software (GraphPad). In all statistical analyses, a p value<0.05 was considered to represent statistically-significant differences.

## Results

### Antisense oligonucleotide-treated, co-stimulation-impaired, immunoregulatory DC (iDC) reverse new-onset hyperglycemia in NOD mice

**Figure 1:**
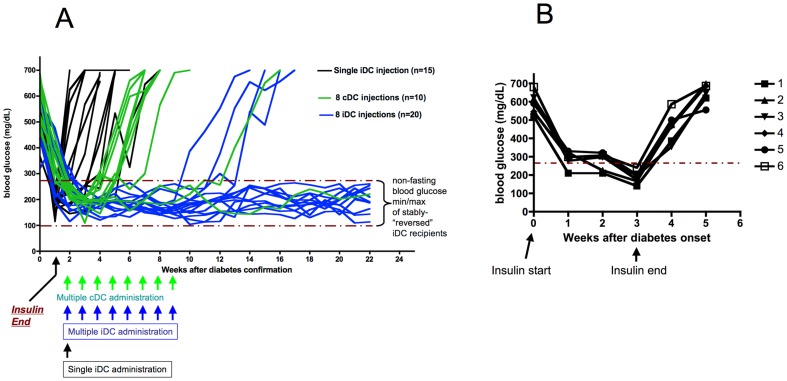
Stable reversal of T1D in NOD mice is achieved by multiple iDC injections. a. Multiple iDC injections (n = 8, blue lines) stably maintain glucose levels within a range between 100–280 mg/dL in most new-onset T1D NOD mice. Multiple control DC injections (n = 8, green lines) reversed T1D in a few recipients, albeit transiently. Single iDC injections (black lines), compared to multiple injections were ineffective in restoration of blood glucose stability. The lines represent non-fasting blood glucose levels in individual NOD mice. Mice were first administered insulin once diabetic hyperglycemia was confirmed until the time glucose levels dropped to below 280 mg/dL (5–8 days on average). At this point, insulin was withdrawn. Then, 2×10^6^ DC (cDC or iDC) were injected s.c at the abdominal flank overlying the gastrointestinal organs. Time 0 represents the time of diabetes confirmation. The black arrow below the x-axis shows the time of insulin withdrawal concomitant with the first DC injection. The coloured arrows show the times at which DC were administered (single or multiple). The dashed lines in the graph indicate the minimum and maximum non-fasting blood glucose levels measured in the reversed iDC treatment group. b. Blood glucose crosses the 280 mg/dL threshold within a day of insulin withdrawal when new-onset diabetic NOD mice (n = 5) are not subjected to any other treatment. These data indicate that insulin administration alone cannot account for the effects of cDC or iDC in prolonging normoglycemia in NOD mouse cohorts shown in Figure 1a.

We compared the efficacy of iDC and cDC to stably restore non-fasting blood glucose to pre-diabetic levels in NOD female mice. In Figure 1a we show that, even though a single iDC injection can transiently reverse new-onset T1D (return to diabetes as early as one week after insulin withdrawal), multiple injections are required for stable, long-term restoration of blood glucose to levels that reflect pre-diabetic conditions (non-fasting glucose levels were, on average, 218+/−63 mg/dL when combining all time points measured; n = 9 NOD mice at 22 weeks). A single cDC injection was ineffective at maintaining non-fasting blood glucose below 280 mg/dL past 12–16 days following the injection (data not shown). Stable restoration of non-fasting glucose levels, in the absence of any exogenous insulin administration, was maintained in all but three iDC recipients for at least 4 months after cessation of iDC administration. Interestingly, multiple cDC injections also exhibited some capacity for T1D reversal (2/10 mice; 20%), although only one of the two reversed mice returned to hyperglycemia by four months after the last cDC injection. Figure 1b shows that blood glucose crosses the 280 mg/dL threshold following insulin withdrawal in new-onset diabetic NOD mice (n = 5) left untreated. Thus, residual insulin from previous administration alone cannot account for the effects of cDC or iDC in stably-restored blood glucose levels in the mice represented in Figure 1a. Furthermore, the mean non-fasting glucose levels at each time point shown in Figure 1a in our iDC-treated cohorts were comparable to those observed in another DC-based reversal approach [67].

### iDC administration into NOD mice induces an increase in the frequency of suppressive CD19+ B220+ CD11c− IL-10+ cells

**Figure 2:**
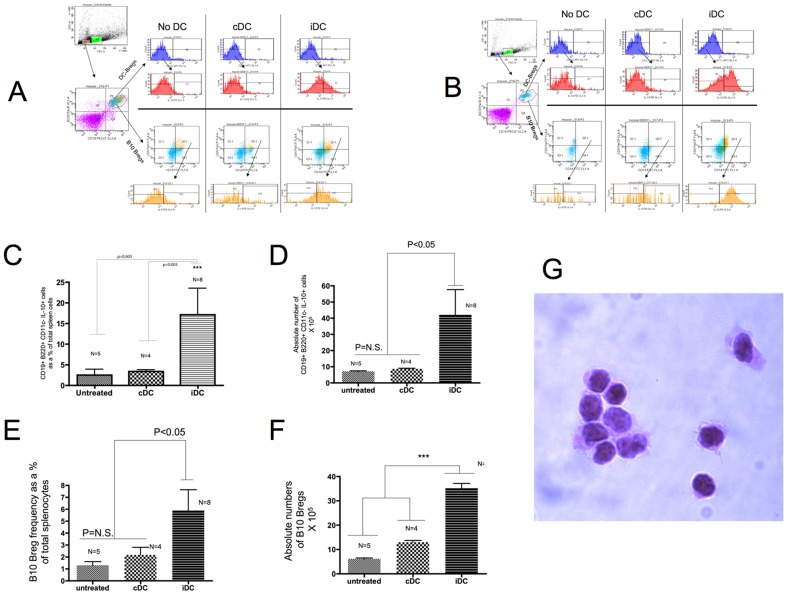
iDC administration into NOD mice promotes an increase in the frequency of CD19+ B220+ CD11c− IL-10+ B-cells as well as B10 Bregs in vivo. 2×10^6^ cDC or iDC were injected into NOD female mice (8–10 weeks) s.c. into the abdominal flank overlying the gastrointestinal organs. Three days later, the spleens were collected and the frequency of the CD19+ B220+ CD11c− IL-10+ B-cells as well as B10 Bregs was measured by flow cytometry. Absolute numbers were calculated based on hematocrit measurements of total viable single cells recovered from the tissue. a. Shown is the flow cytometric approach used to identify and measure the frequency of the CD19+ B220+ CD11c− IL-10+ cells (DC-Bregs; top two histograms) and B10 Bregs (bottom two histograms). The results shown are representative of analyses conducted in freshly-collected splenocytes of at least four different NOD recipients of each type of DC population (and PBS vehicle control). b. The data are representative of analyses conducted in freshly-collected pancreatic lymph node single cells acquired from the same mice (n = 4 per treatment type) referred to above in (a). c. The graph summarises the frequency of CD19+ B220+ CD11c− IL-10+ B-cells (DC-Bregs) measured by flow cytometry as a % of total splenocytes. The number of mice from which tissue was collected per treatment type is shown at the top of the bars which represent the median value. The error bars reflect the SD. The differences between iDC and cDC/control untreated were statistically-significant (p<0.005, Kruskal-Wallis test of variance). d. The graph summarises the absolute number of DC-Bregs measured by flow cytometry in the freshly-collected spleens of the untreated, cDC and iDC-injected NOD mice (same mice as in [c] above). The bars represent the medians and the error bars the SD. The differences among the medians was statistically-significant as shown in the graph (Kruskal-Wallis test of variance). e. The graph summarises the frequency of B10 Bregs (CD19+ CD1d+ CD5+ Il-10+ cells) measured by flow cytometry as a % of total splenocytes. The number of mice from which tissue was collected per treatment type is shown at the top of the bars which represent the median value. The error bars reflect the SD. The differences between iDC and cDC/control untreated were statistically-significant (p<0.05, Kruskal-Wallis test of variance). f. The graph summarises the absolute number of B10 Bregs measured by flow cytometry in the freshly-collected spleens of the untreated, cDC and iDC-injected NOD mice (same mice as in [e] above). The bars represent the medians and the error bars the SD. The differences among the medians was statistically-significant (p<0.005, Kruskal-Wallis test of variance). g. Hematoxylin/Eosin-stained cytospin of flow-sorted B10 Bregs. Cells were sorted from freshly-isolated splenocytes of a 10 week-old female non-diabetic NOD mouse. Magnification ×63. The morphology is identical among cytospins from another 5 age-matched female NOD mice.

Our findings in humans suggested that iDC increase the frequency of suppressive B-cells [57]. Since then, we have shown that exposure of human B-cells to iDC in culture results in proliferation of existing Bregs [61]. We therefore hypothesised that, in addition to the involvement of CD4+ CD25+ T-cells [23], part of the beneficial results of iDC-induced reversal of T1D in NOD mice could involve an iDC-induced expansion of suppressive B-cells, including CD1d+ CD5+ Bregs. To address this hypothesis, non-diabetic female NOD mice (8–18 weeks of age) were given a single injection of cDC or iDC. When mice were administered cDC, we observed a slight increase in IL-10+ cells inside a population of CD19+ B220+ CD11c− cells in spleen (Figure 2a), although compared to untreated control recipients, this difference was not statistically-relevant. In contrast, iDC recipients exhibited a substantial and statistically-significant increase in the frequency and number of these B-cells (Figure 2a and summarised in the graphs in Figure 2c). CD1d+ CD5+ cells inside the population of IL-10+ cells also increased slightly in frequency in cDC recipients although at a statistically-insignificant level, but the increase in iDC recipients was remarkable and statistically-relevant when compared to cDC and control-treated recipients. The same effect was observed when single cells from pancreatic lymph nodes were examined (Figure 2b) Figures 2c–2f summarise the differences in the frequency and actual numbers of splenic CD19+ B220+ CD11c− IL-10+ B-cells as well as B10 Bregs in untreated, cDC and iDC-administered NOD mice. B10 Bregs have never been observed microscopically. For the first time, we show these cells under high-power magnification in Figure 2g. These cells were flow-sorted to purity from freshly-collected splenocytes of a 10 week old NOD female mouse. Their morphology was consistent when sorted from five different age-matched females. Even though these B-cells are detectable by flow cytometry in cells from pancreatic lymph nodes (PLN), they are very low in numbers. This limitation, along with the concern that pooling of PLN-cells from multiple mice could yield confounding data, impeded our desire to identify absolute cell number differences in PLN of cDC/iDC/untreated mice.

**Figure 3:**
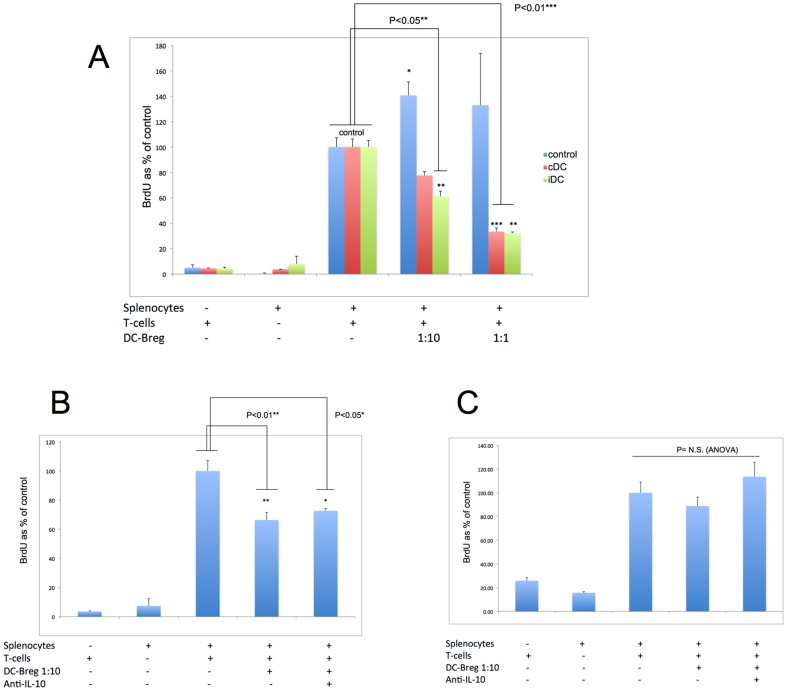
CD19+ B220+ CD11c− IL-10+ B-cells (DC-Bregs) are functionally-suppressive in allogeneic MLR *in vitro* and do not require IL-10 to mediate suppression. a. Quintuplicate wells of splenic T-cells, irradiated splenocytes (alone, together, or in the presence of CD19+ B220+ CD11c− IL-10+ B-cells) were incubated for 5 days. On the last day, BrdU was added. The number of BrdU+ cells was measured by flow cytometry on day 6. Cultures consisted of 1×10^5^ T-cells from the spleen of NOD female mice (8 weeks), irradiated allogeneic splenocytes (C57BL/6 males, 8 weeks) and purified CD19+ B220+ CD11c− IL-10+ B-cells (combination of column enrichment and flow sorting as described in the Methods). Proliferation of only T-cells or splenocytes was taken to represent 100% proliferation in these analyses. The bars represent the mean of n = 5 wells and the error bars represent the SEM. The differences in proliferation in co-cultures of CD19+ B220+ CD11c− IL-10+ B-cells in the absence of cDC or iDC compared to those in the presence of the DC are statistically-significant (p values shown in graph on top of bars as single, double (p<0.05, ANOVA) or triple asterisks (p<0.01, ANOVA). The last two sets of bars compare the proliferation fo the T-cells at a 1∶10 and a 1∶1 ratio of DC-Breg: T-cell numbers. b. CD19+ B220+ CD11c− IL-10+ B-cells (DC-Bregs) are suppressive in allogeneic MLR even in the presence of a neutralising IL-10 antibody but not when physically separated from the T-cells/antigen-presenting cells. Quintuplicate wells of splenic T-cells, irradiated splenocytes (alone, together, or in the presence of DC-Bregs) were incubated for 5 days. The ratio of DC-Breg∶T-cell numbers was 1∶10 in all co-cultures. On the last day, BrdU was added. Anti-IL-10 antibody was added at 1 microgram/mL where shown. Proliferation of T-cells in the presence of irradiated splenocytes was taken to represent 100% proliferation. The bars represent the means of BrdU+ cells as a % of BrdU+ in the control T-cells∶splenocyte co-cultures (n = 5 wells) and the error bars represent the SEM. The differences in T-cell proliferation in co-cultures in the absence of DC-Bregs and those in the presence of DC are statistically-significant (p values shown in graph, ANOVA). c. DC-Bregs cannot suppress the proliferation of Transwell barrier-sequestered T-cells in allogeneic MLR *in vitro*. MLR was conducted with DC-Bregs added on top of a Transwell insert separating co-cultures of T-cells and allogeneic irradiated splenocytes. The ratio of DC-Breg∶T-cell numbers was 1∶10 in all co-cultures Anti-IL-10 antibody was added at 1 microgram/mL where shown on top of the Transwell insert (with the DC-Bregs). Proliferation of cells in the bottom of the dish (T-cells∶splenocyte co-cultures) in the absence of DC-Bregs was taken to represent 100% proliferation in these analyses. N = 5 wells for each co-culture shown. N.S. = differences among means were not significant (p>0.05).

To ascertain if the DC-responsive, splenic CD19+ B220+ CD11c− IL-10+ B-cells represented, or contained Breg populations, we performed an allogeneic MLR assay. Freshly-collected splenocytes from iDC-injected NOD female mice, first enriched into IL-10-producing cells and then flow sorted into a CD19+ B220+ CD11c− population, were added at a 1∶10 and 1∶1 ratio to syngeneic splenic T-cells and allogeneic irradiated splenocytes. In Figure 3a we show that at a B-cell∶T-cell ratio of 1∶10, the CD19+ B220+ CD11c− IL-10+ B-cells isolated from both the cDC and iDC-treated NOD mice *were* sufficient to significantly reduce T-cell proliferation by at least 50%. At the higher 1∶1 ratio, this B-cell population isolated from both cDC and iDC NOD recipients significantly reduced T-cell proliferation by ≈67% (Figure 3a). The addition of an IL-10 neutralising antibody was unable to prevent the suppression of T-cell proliferation in the presence of these B-cells (Figure 3b). Furthermore, the physical separation of these B-cells from the allogeneic MLR co-culture by Transwell barrier resulted in the loss of suppression of T-cell proliferation (Figure 3c). These data suggest that CD19+ B220+ CD11c− IL-10+ B-cells represent a population of suppressive B-cells where IL-10, even if produced, may not be required for their suppressive ability, at least *in vitro*. We tentatively assign the term “DC-Bregs” to this B-cell population, given their response to iDC.

### cDC and iDC induce proliferation of DC-Bregs and convert B-cells into IL-10-expressing DC-Bregs in vitro

**Figure 4:**
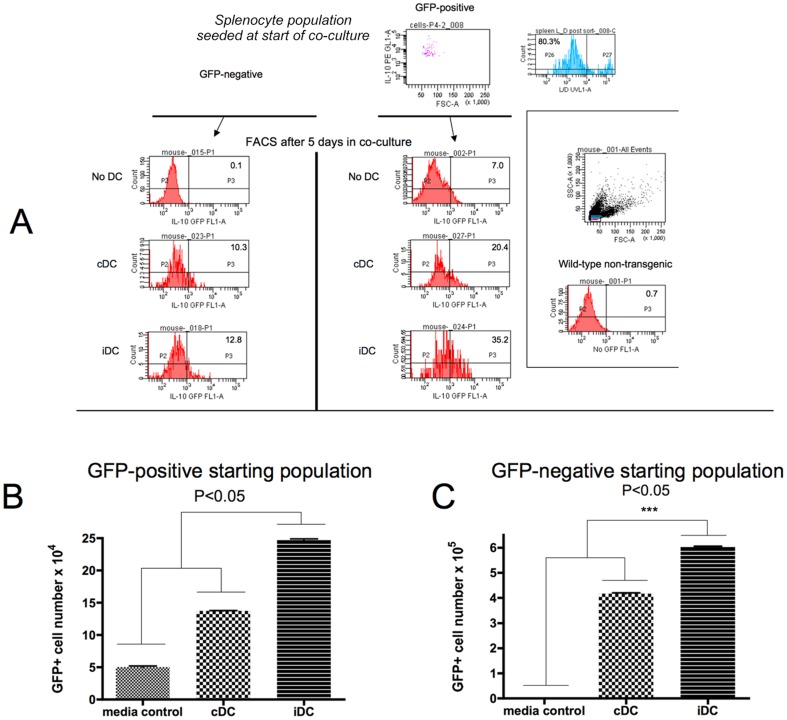
cDC and iDC promote proliferation of DC-Bregs as well as conversion of IL-10− into IL-10+ DC-Bregs *in vitro*. a. Freshly-collected splenocytes from IL10^gfp^ transgenic mice were flow sorted into CD19+ B220+ CD11c− GFP+ or CD19+ B220+ CD11c− GFP− populations with very stringent gating to exclude autofluorescent cells based on the fluorescence characteristics of flow-sorted CD19+ B220+ CD11c− cells from freshly-collected splenocytes of the wild-type mouse strain (far right panel inset). Purity of the GFP+ after sorting (indicated as IL-10 versus FSC in the top most quadrant plot) as well as cell viability (Live/Dead staining in the histogram adjacent to the top most quadrant plot), representative of all the sorting outcomes performed in this experiment is shown. 5×10^4^ sorted cells (GFP+ or GFP−) were placed into co-culture with PBS, or an equal number of cDC or iDC. Representative GFP fluorescence of the B-cells after 5 days in co-culture with cDC, iDC and media is shown in the histograms with the GFP+ cells represented as a % of total cells in culture (values of this specific experiment, representative of three separately-conduced experiments, are shown inside the histograms). b. The graph shows the actual number of GFP+ DC-Bregs *in vitro* after co-culture of a highly-purified GFP− starting population (5×10^4^ cells) with media, cDC or iDC. The bars indicate the mean of triplicate wells and the error bars the SEM. p<0.05 shown by asterisk (ANOVA). c. The graph shows the actual number of GFP+ DC-Bregs *in vitro* after co-culture of a highly-purified GFP+ starting population (5×10^4^ cells) with media, cDC or iDC. The bars indicate the mean of triplicate wells and the error bars the SEM. p<0.05 shown by asterisk (ANOVA).

To determine if the increase in DC-Bregs in DC recipients is a result of a DC-induced proliferative signal to existing DC-Bregs or a consequence of differentation of CD19+ B-cells into IL-10-producing cells, we co-cultured CD19+ B220+ CD11c− B-cells flow sorted from freshly-isolated spleen of transgenic mice expressing the GFP reporter from the IL-10 promoter (IL10^gfp^ mice) with cDC or iDC generated from the wild-type, non-transgenic, C57BL/6 strain. At the same time, to determine if DC caused CD19+ B220+ CD11c− B-cells to acquire GFP (IL-10) expression (i.e. a differentiation/conversion mechanism) we co-cultured flow-sorted CD19+ B220+ CD11c− GFP-negative cells with the cDC, iDC or alone. In all instances, the sorted cells were >80% viable at the onset of culture (top most histogram, Figure 4a). The GFP+ sorted cells were routinely >95% pure (top most histogram, Figure 4a) upon culture initiation. After 5 days in culture, we measured the frequency of GFP+ cells by flow cytometry (representative experimental outcome shown Figure 4a). Figures 4b and 4c summarise the outcome of these co-culture experiments on an absolute cell number basis and show that cDC and iDC induced an increase in GFP+ cell number when the sorted input B-cells were GFP+ (Figures 4b and 4c) reflecting proliferation *in vitro*. cDC and iDC also induced an increase in the GFP+ cell number when the input B-cells were GFP− (Figures 4d and 4e), reflecting a concomitant conversion mechanism. Viability of cells at the end of the 5 days was no less than 80% (data not shown). When comparing the actual number of GFP+ cells in the co-cultures at the end of the experiments, it becomes evident that both proliferation and differentiation events co-incide in the presence of cDC and iDC, with the effects of iDC greater in magnitude than cDC (Figure 4b compared to Figure 4c).

### B10 Bregs are the majority B-cell constituent of the suppressive DC-Bregs

**Figure 5:**
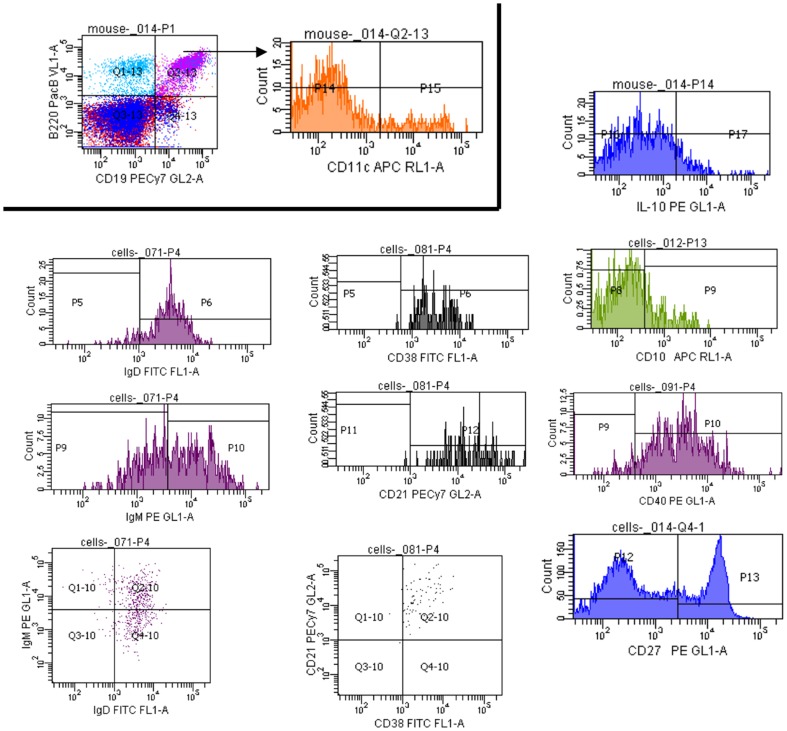
Flow cytometric characterisation of splenic DC-Bregs from NOD mice. Gating was established as shown (CD19+ B220+ CD11c−). The frequency of cells expressing each of the surface proteins indicated in the histograms inside this gate further established the phenotype of the DC-Bregs in freshly-isolated spleen of NOD female mice (10 weeks of age). The data shown are representative of the flow cytometric anlyses of freshly-acquired splenocytes from three different age-matched NOD mice. Surface markers include IgD, IgM, CD10, CD21, CD27, CD38 and CD40.

CD19 and B220 co-positivity reflects a heterogeneous leukocyte population enriched in B-cells, thus, DC-Bregs are expected to represent a wide array of B-cells at different stages of differentiation and of variable function. We considered it highly-unlikely that every single cell in this population was inherently suppressive. In order to identify the suppressive constituent population(s) and to determine if cDC/iDC affected these populations specifically, in the same manner as they affected the heterogeneous DC-Breg cells, we first phenotypically-characterised the DC-Bregs. We first focused on cell surface markers associated with recently-reported mouse and human Bregs as well as other cell surface markers that could give an insight into their ontogeny and their mechanisms of action. Figure 5 shows the outcome of a representative flow cytometric characterisation of NOD DC-Bregs enriched into CD19+ B220+ CD11c− cells from freshly-collected splenocytes. Although we did not exhaustively determine the expression of all possible B-cell markers, the NOD DC-Bregs expressed surface IgD and IgM, CD10, CD21, CD27 and CD38. Interestingly, the DC-Bregs also expressed CD40. Based on the marker presence and the mean fluorescence intensity, we tentatively consider DC-Bregs to be CD19+ B220+ CD11c−, IgD^HIGH^, IgM+, CD10^LOW^, CD21+, CD27+, CD38+, CD40^HIGH^, IL-10+, suggesting the possible presence of at least one memory B-cell population (based on the presence of CD19+ CD27+ CD38+ CD40+; [68], [69]). This memory subpopulation may be resting/proliferating and may or may not represent suppressive cells. These are questions that we are currently addressing.

**Figure 6:**
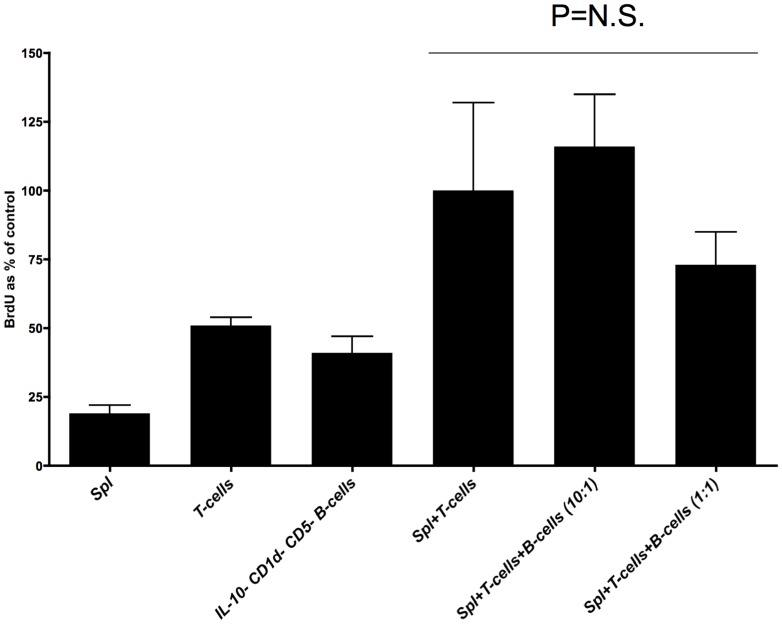
B10 Breg-depleted DC-Bregs are not suppressive in allogeneic MLR *in vitro*. The graph shows the frequency of BrdU-positive (proliferating) T-cells from freshly-isolated spleen of IL10^gfp^ transgenic mice in the presence or absence of syngeneic CD1d, CD5, IL-10-depleted B-cells (represented in the graph as “IL-10− CD1d− CD5− B-cells”) and allogeneic, irradiated splenocytes (Spl). The B-cells (DC-Bregs or B10 Breg-depleted DC-Bregs) were added at a 1∶1 or a 1∶10 ratio of B-cells∶T-cells. The frequency of BrdU+ cells was measured by flow cytometry and the data are shown as BrdU+ cells as a % of control, where control refers to the frequency of proliferation of T-cells in the presence of only irradiated allogeneic splenocytes (taken to be 100%). The bars represent the mean and the error bars the SEM (triplicate culture dish wells). The differences in proliferation of T-cells among the co-cultures were not statistically-significant (p = 0.118, ANOVA) The data are representative of two independently-conducted experiments.

At the same time, it was apparent that the DC-Bregs also exhibited features reminiscent of B10 Bregs [55]. We thus sought to determine if B10 Bregs were the sole, or at least one of the suppressive populations comprising the DC-Bregs. Using the IL10^gfp^ transgenic strain as a source of B-cells we discovered that B10 Breg-depleted DC-Bregs (i.e. the cells that remain in the effluent as IL-10− CD1d− CD5− CD19+ B220+ CD11c− cells during flow sorting of freshly-collected splenocytes into B10 Bregs) exhibit a trend of suppressive ability, however, on a statistical basis there were no differences among co-cultures (Figure 6).

### B10 Bregs express RA receptors

**Figure 7:**
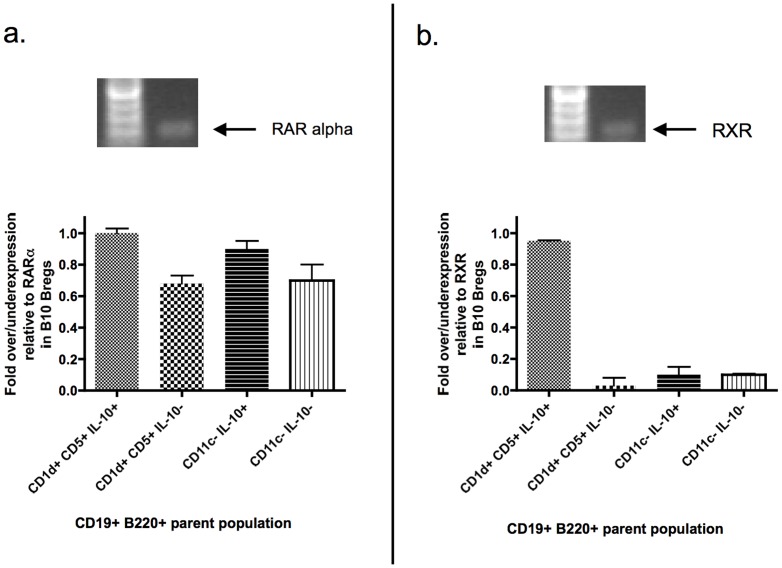
DC-Bregs and B10 Bregs express RA receptors. Real-time, semi-quantitative RT-PCR confirms the presence of steady-state mRNA of RA receptor alpha (Figure 7a) as well as low levels of retinoid X receptor (Figure 7b) in highly-purified (flow-sorted) DC-Bregs and B10 Bregs from freshly-isolated spleen of IL10^gfp^ transgenic mice. The gel images in Figure 7a and 7b show the RT-PCR products from flow-sorted B10 Bregs. The steady-state mRNA levels of RARalpha and RXR from the B10 Bregs were used as the controls and the values were taken to represent 100% receptor expression. Steady-state mRNA levels of RARalpha and RXR in non-B10 Breg populations are shown in the graphs underneath the gel images normalised to the B10 Breg value and presented as fold under or overexpression. Steady-state RARalpha mRNA was detected in B10 Bregs (first bar in graph; leftmost)), IL-10− CD19+ CD5+ CD1d+ cells (second bar in graph), DC-Bregs (third bar in graph), and IL-10− DC-Bregs (last bar in graph; rightmost). The bars represent the medians and the error bars the SD. These data are representative of steady-state mRNA from flow sorted cells from two different spleens of age-matched mice (10 week-old females).

Given the data demonstrating that B10 Bregs account for most of the suppressive activity in the DC-Breg population, we decided to focus on the B10 Bregs from this point onward. Indirect observations suggest that B-cells accumulating into inflamed intestinal tissue share features with Bregs [70]. RA appears to be one of the molecules that direct such homing [70]. RA is also central in the differentiation of T-cells into Foxp3+ Tregs [71]. Furthermore, we have recently demonstrated the highly-purified human CD19+ CD24+ CD38+ Bregs express RA receptor alpha and are response to RA *in vitro* [72]. To determine if B10 Bregs are formally able to respond to RA, we performed real-time, semi-quantitative RT-PCR on IL10^gfp^-derived flow sorted B10 Bregs for RA receptor (RAR) isoforms. In Figure 7 we show that B10 Bregs as well as DC-Bregs (represented as CD11c− IL-10+ in the graphs) express RAR and the retinoid X receptor (RXR).

### Diabetes-suppressive DC produce RA

**Figure 8:**
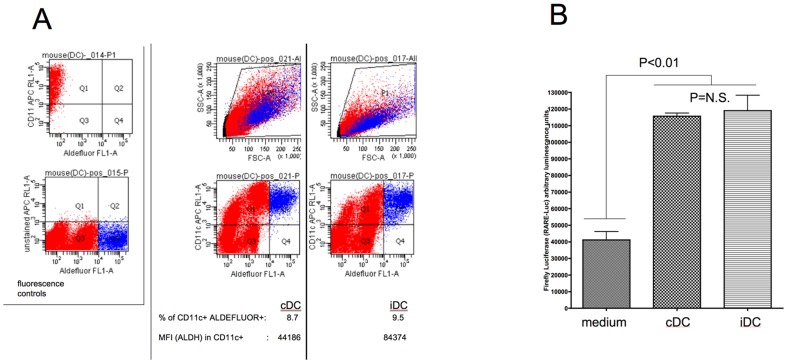
cDC and iDC produce bioactive RA *in vitro*. a. The ALDEFLUOR reagent stains aldehyde dehydrogenase-expressing cells and thus RA-producing cells. Even though cDC are ALDEFLUOR+, and the frequency of CD11c+ ALDEFLUOR+ cDC is similar to CD11c+ ALDEFLUOR+ iDC (cDC: 8.7% cells are CD11c+ ALDEFLUOR+ compared to 9.5% of iDC), on a per-cell basis (Mean Fluorescence Intensity; MFI) iDC are twice as reactive with ALDEFLUOR than cDC (44186 compared to 84374). The flow cytometric analysis shown is representative of duplicate cultures of four age- and sex-matched NOD female mice (7 weeks of age). The two quadrant plots at the far left of the graph, in the inset, demonstrate the compensation performed for the flow cytometry; there was no non-specific fluorescence (“bleeding”) into either of the measured channels. b. RA-response element (RARE)-driven luciferase activity is detectable in RARE-Luc plasmid-transduced HEK293 cells cultured in the presence of cDC or iDC where DC were placed on top of a Transwell barrier separating them from the RARE-Luc-transduced 293 cells. Luciferase activity was measured after 24 hours culture. The bars represent the mean relative luminescence (arbitrary units) of triplicate cultures and the error bars represent the SEM. The differences between the cDC and iDC means were not significant (Student's t-test). HEK293 cells were co-transfected with a CMV-Renilla luciferase control plasmid concurrently with the RARE-Luc (Firefly) to control for transfection efficiency. There were no differences in transfection efficiency as indicated by statistically-indistinguishable Renilla activity among all cultures (not shown).

Affymetrix expression technology showed that human cDC and iDC as well as cDC and iDC generated from NOD mouse bone marrow progenitors express aldhehyde dehydorgenases 1 and 2 (ALDH), rate-limiting enzymes for RA biosynthesis [65], [66], [72]. iDC in particular expressed significantly greater steady-state amounts of the 1A2 isoform associated with tolerogenic DC [73] compared to cDC (data not shown). In Figure 8a we demonstrate that cDC and iDC produce RA. ALDEFLUOR conversion to fluorescent product indicates that ALDH is enzymatically-active in these cells. The frequency of ALDH+ DC as a percentage of gated cells in cDC and iDC is similar. However, on a per cell basis (reflected in the geometric mean fluorescence intensity; Figure 8a), iDC are twice as reactive with the ALDEFLUOR reagent compared to cDC. This may reflect more ALDH enzyme levels per cell, a faster catalytic rate for RA biosynthesis or both. In Figure 8b we confirm that cDC and iDC produce bioactive RA. By 24 hours, however, the concentration of bioactive RA produced by both cDC and iDC was at levels able to comparably stimulate RA-dependent reporter gene activity *in vitro* (Figure 8c).

## Discussion

Despite convincing arguments supporting the consideration to use tolerogenic DC in human disease and/or transplantation [26] no attempt had been made until 2007. Our group was the first to test the safety of autologous co-stimulation-impaired DC in established T1D patients. The outcome was reported in 2011 [57]. Those DC are essentially identical to the ones described herein. Both the human and the murine DC are generated in the presence of a mixture of phosphorothioate DNA oligonucleotides targeting the primary transcripts of CD40, CD80 and CD86. One novel and intriguing outcome of the clinical trial was the increased frequency of B-cells presenting an immunosuppressive character *in vitro*, particularly in iDC recipients. This prompted us to investigate a mechanistic link between our tolerogenic DC and immunosuppressive B-cells. We have recently shown that human iDC induce the differentiation of human B-cells into suppressive B-cells *in vitro* [61] establishing for the very first time a link between tolerogenic DC and suppressive B-cells, potentially Bregs.

Although production of IL-10 appears to be characteristic of all Bregs identified to date, the surface phenotype of the different populations described is very variable. Some studies have identified suppressive acitivty in CD5+ B1a cells, CD21+ CD23− marginal zone cells, or CD1d+ CD21+ CD23+ T2-marginal zone precursor B-cells [74]. In humans, the suppressive activity seems to concentrate inside CD19+ CD24+ CD27+ CD38+ cells [30], [56]. Production of IL-10 however is not necessarily a condition for B-cells, including Bregs, to confer suppression - at least *in vitro* - and not all IL-10-producing B-cells are necessarily regulatory [75]. Accumulating evidence suggests a model where IL-10 expression may be transient as B-cells with suppressive potential could transition through an IL-10-expressing phase. These cells come to rest as immunoglobulin-secreting cells with suppressive capacity, although they are not solely, or completely reliant on IL-10 for suppressive capacity [76]. Based on these observations, models have been proposed where Bregs, including the B10 population, emerge from a transitional and/or memory population consequent to antigen exposure and BCR activation. The same models predict that the Breg BCR repertoire is polyclonal.

Based on such observations, we propose that B-cells, in general, have an intrinsic potential to differentiate or to transition through states where they can suppress the proliferation of other B-cells as well as T-cells, but that this potential is only realised and activated in response to microenvironmental signals including those from tolerogenic DC. Given the requirement to provide CD40 stimulation for Bregs to expand [52], [76], [77], [78], one potential signal concurrent with BCR activation could be CD40 Ligand. BCR-activated B-cells produce IL-10 and their adoptive transfer into NOD mice reduced disease incidence and severity [79]. CD40 Ligand is expressed on TLR9-activated myeloid DC [80], as well as on activated CD4+ T-cells [81], [82] which operate through B∶T-cell interactions [83], [84], [85]. When considering the interplay among DC∶T-cells∶B-cells in the context of CD40 Ligand and microenvironmental influences through cytokines, including IL-21, recently shown to induce significant expansion of B10 Bregs [86], one could appreciate why the suppressive potential of B-cells, even though described more than three decades ago [87], [88], eluded many investigators.

Modulation of B-cell frequency as an approach to attenuate autoimmunity is not a new concept. Many studies have shown that B-cell depletion ameliorates, prevents and in some instances can reverse autoimmunity [45], [89], [90], [91], [92], [93], [94], [95]. Mechanistic insights indicate anergy and immune deviation along with an increased threshold for antigen presentation as operationally-underlying the disease prevention and improvement [45], [89], [90], [91], [92], [93], [94], [95]. Accumulating data, however, strongly suggest that B-cell depletion is followed by a period of homeostatic expansion of B-1 type, transitional B-lymphocytes, and their progenitors [96], [97], [98], [99] and the surface phenotypes of those cells are strongly suggestive of B-cells with a regulatory, immunosuppressive ability [97], [98], although this has not been formally demonstrated. Indeed, such a mechanism, involving homeostatic expansion of Bregs secondary to B-cell depletion could underlie and partially explain the successful outcomes in islet allotransplantation and reversal of T1D in NOD mice following B-cell deletion using B-cell targeted antibodies [44], [45], [46], [47].

The data shown in Figure 4 raise the question of why the frequency of B-cells not expressing GFP from the IL-10 promoter was considerably lower, in contrast to the higher frequency of GFP-expressing cells when co-cultured in the presence of cDC/iDC (iDC effects>cDC; Figure 4). One possible interpretation is that immature DC (cDC) and tolerogenic DC confer increased survival (i.e. increased anti-apoptotic states) selectively to suppressive B-cells, and/or induce apoptosis selectively in non-suppressive B-cells. These two potential mechanisms need not be mutually-exlusive. Since B10 Bregs produce IL-10, and their frequency is increased in response to tolerogenic DC, this IL-10 could act in an autocrine manner as well as in a paracrine fashion to bolster regulatory cell networks that rely on IL-10 for their function. IL-10 is a well-known survival factor for many cell types including B-cells (depending on their state of activation; [100], [101]). We speculate that there are differences in IL-10 receptor levels and possibly signaling among B10 Bregs, DC-Bregs and non-suppressive B-cells. Furthermore, we also speculate that receptors for possibly other pro-survival signals are preferentially-expressed on the cell surface of suppressive B-cells compared to non-suppressive cells. We are currently studying these possible mechanisms.

Our recent findings in human cells showing a potential role for RA in suppressive B-cell biology [72] are now also relevant at least for mouse B10 Bregs. RA, a molecule relevant in tolerance mechanisms, could be one of a number of soluble factors that condition or confer fitness to B-cells to assume and to stabilise a suppressive ability. RA-producing DC are immunosuppressive and therapeutic in animal models of inflammatory bowel disease and their efficacy rests on RA-mediated differentiation of Foxp3+ Tregs [57]. We now add suppressive B-cells/Bregs to the list. RA production by iDC can explain, in part, our observations of increased CD4+ CD25+ T-cell frequency in successfully-treated NOD mice [23] as well as the increased frequency of suppressive B-cells, which express, as we have shown herein, RA receptors. The seminal findings of Clare-Salzer and colleagues [102], who showed that B-cells were increased in frequency in NOD mice injected DC from syngeneic pancreatic lymph nodes, could represent an a process of DC-induced suppressive B-cell expansion, as part of a therapeutic mechanism as they originally reported.

Other groups who have studied Bregs to date, often prestimulate the cells with recombinant CD40L and/or PMA/ionomycin [10]. We chose to avoid these *in vitro* manipulations primarily because such treatments could potentialy alter the natural phenotype and activity of freshly-acquired Bregs.

In summary, we have discovered that tolerogenic DC engineered ex vivo for low co-stimulation ability increase frequency and numbers of IL-10-expressing B-cells in vitro and in vivo, consequent to concomitant proliferation of pre-existing IL-10+ B-cells and conversion of CD19+ B-lymphcytes into IL-10-expressing cells without affecting their immunosuppressive potential in vitro. We also discovered that these DC-sensitive putative Bregs expressed receptors for retinoic acid which is produced by the tolerogenic dendritic cells. Taken together, our findings usher a potential new therapeutic approach to T1D immunotherapy which involves suppresisve B-cells, possibly homogeneous Bregs, together with approaches and molecules that stabilise their suppressive ability and expand their numbers *in vitro* (e.g. RA, IL-10, IL-21 [86]).
